# Basal metabolic rate and maternal energetic investment durations in mammals

**DOI:** 10.1186/s12862-014-0194-z

**Published:** 2014-09-14

**Authors:** Gabrielle Jackson, Arne Ø Mooers, Evgenia Dubman, Jenna Hutchen, Mark Collard

**Affiliations:** Human Evolutionary Studies Program, Simon Fraser University, 8888 University Drive, Burnaby, BC V5A 1S6 Canada; Department of Archaeology, Simon Fraser University, 8888 University Drive, Burnaby, BC V5A 1S6 Canada; Department of Biological Sciences, Simon Fraser University, 8888 University Drive, Burnaby, BC V5A 1S6 Canada; Department of Archaeology, University of Aberdeen, St Mary’s Building, Elphinstone Road, Aberdeen, AB24 3FX UK

## Abstract

**Background:**

The Metabolic Theory of Ecology (MTE) predicts that gestation duration, lactation duration, and their sum, total development time, are constrained by mass-specific basal metabolic rate such that they should scale with body mass with an exponent of 0.25. However, tests of the MTE’s predictions have yielded mixed results. In an effort to resolve this uncertainty, we used phylogenetically-controlled regression to investigate the allometries of gestation duration, lactation duration, and total development time in four well-studied mammalian orders, Artiodactyla, Carnivora, Primates, and Rodentia.

**Results:**

The results we obtained are not consistent with the predictions of the MTE. Gestation duration scaling exponents are below 0.25 in all four orders. The scaling exponent for lactation duration is below 0.25 in Carnivora and Rodentia, indistinguishable from 0.25 in Artiodactyls, and steeper than 0.25 in Primates. Total development time scales with body mass as predicted by the MTE in Primates, but not in artiodactyls, carnivores, and rodents. In the latter three orders, the exponent is 0.15.

**Conclusions:**

Together, these results indicate that the influence of basal metabolic rate on mammalian maternal investment durations must be more complicated than the MTE envisages, and that other factors must play an important role. Future research needs to allow for the possibility that different factors drive gestation duration and lactation duration, and that the drivers of the two durations may differ among orders.

**Electronic supplementary material:**

The online version of this article (doi:10.1186/s12862-014-0194-z) contains supplementary material, which is available to authorized users.

## Background

Mammalian species vary markedly in the length of the key phases of the maternal investment cycle—gestation and lactation [1–3]. Phylogenetic inertia and differences in body mass explain some of the interspecific variation, but it is clear that other factors must also be involved. What these factors are has been a matter of debate for over 30 years.

Conventionally, researchers have treated gestation and lactation as unrelated variables and developed separate hypotheses to account for the inter-specific variation in the duration of each [2,4–10]. In contrast, Hamilton et al. [11] have recently argued that the allometric scaling of both durations and their sum, which Hamilton et al. [11] call “total development time,” is consistent with the predictions of the Metabolic Theory of Ecology (MTE). A negative-quarter power scaling of mass-specific basal metabolic rate (BMR) with body mass is expected under the MTE. Because biological time periods are expected to scale with the inverse of mass-specific metabolic rate, the MTE predicts that gestation duration, lactation duration, and total development time should scale allometrically with a slope of 0.25 [12].

The MTE approach to explaining the allometries of mammalian gestation duration, lactation duration, and total development time is elegant, but empirical research does not unambiguously support its predictions. A number of early studies of the scaling of maternal investment durations with body size returned exponents consistent with the MTE (e.g. [13,14]), but several others found the relevant exponents to be markedly lower than 0.25 (e.g. [15,16]). Recently, Hamilton et al. [11] tested predictions of the MTE with data from >4000 placental and marsupial mammal species and ordinary least-square (OLS) regression. They found that the allometries for gestation duration, lactation duration, and total development time were indistinguishable from the MTE predicted value of 0.25 in placental mammals. They also found that marsupial lactation duration scaled with body mass with the predicted exponent. However, gestation duration and total development time in marsupials scaled with body size with much lower exponents than predicted by the MTE (0.04 and 0.17, respectively). Dubman et al. [17] investigated whether the MTE can explain the variation of gestation duration, lactation duration and total development time in primates. They found that total development time scales as predicted by the MTE, but gestation duration and lactation duration do not. Specifically, gestation duration scales with a significantly lower exponent than the MTE predicts, while lactation duration scales with a significantly higher exponent than the MTE predicts. Most recently, Clauss et al. [18] used a sample of 1214 species from 20 orders to examine whether gestation duration in eutherian mammals scales in manner predicted by the MTE. They found that gestation duration scales with an allometric slope of 0.25 but only when OLS regression was used. When phylogenetically-correlated error variance was incorporated into modeling, the allometric slope of gestation duration was much shallower and consistent with that reported by Dubman et al. [17] for primates.

Currently, it is not clear how to explain the fact that some empirical studies support the MTE’s predictions regarding the scaling exponent for gestation duration, lactation duration, and total development time, while others do not. It could be due to methodological differences, but differences in taxonomic coverage might also be responsible. In an effort to resolve this uncertainty and therefore clarify whether or not the MTE explains maternal energetic investment durations in mammals, we used phylogenetically-controlled regression to examine the scaling of gestation duration, lactation duration, and total development time within and across four orders of mammals—Artiodactyla^a^, Carnivora, Primates, and Rodentia. We selected these orders because they span a wide range of body sizes, and have relatively good life history data and reasonably well resolved phylogenies. In addition, two of them are dominated by altricial species (Carnivora, Rodentia) while the other two comprise mostly precocial species (Artiodactyla, Primates); the altricial-precocial axis is known to be associated with gestation duration [18,19].

## Methods

The dataset comprised species-mean values for four variables for 457 species. The variables were adult body mass (in grams), gestation duration (in days), lactation duration (in days), and total development time (in days). Mass-specific BMR (in ml O2/hr/g) was also included when available, which was the case for 149 of the 457 species.

Values for adult body mass, gestation duration, lactation duration, and BMR were obtained from the PanTHERIA database [20]. We opted to use the PanTHERIA database after Hamilton and colleagues turned down our request for a copy of the Hamilton et al. [11] dataset. PanTHERIA’s body mass data are derived from captive and/or wild individuals who were non-gravid and live or freshly killed at the time of measurement. PanTHERIA defines gestation duration as the length of time of non-inactive fetal growth. Lactation duration is called “weaning age” in PanTHERIA, and is defined as the age when primary nutritional dependency on the mother ends and independent foraging begins to make a major contribution to the offspring’s energy requirements. PanTHERIA’s BMR values are derived from wild and/or provisioned individuals of known body mass. The individuals were resting and not subject to thermoregulatory stress when BMR was measured. Total development time was calculated by summing gestation duration and lactation duration. Mass-specific BMR was computed from two of the variables provided by PanTHERIA: total BMR and body mass of the individual(s) whose total BMR was measured.

All artiodactyl, carnivore, and primate species with both lactation and gestation data in PanTHERIA were included in the sample. Rodentia has many more species with available data than the other orders (n = 296). Consequently, we only included some rodent species in our sample. Rodent species were selected so that all families with available data were represented and body size variation was maximized. Of the 457 species in the sample, 100 are artiodactyls, 146 carnivores, 106 primates, and 105 rodents.

We began by checking that the sample was suitable to test the predictions of the MTE hypothesis. This was accomplished by regressing the log of mass-specific BMR on the log of body mass (n = 149) using the phylogenetic generalized least squares (PGLS) method and the ape and nlme packages [21,22] in R version 2.15.1 [23]. The phylogenetic parameter λ was estimated separately for each model [17]. The trees used for phylogenetic correction were obtained by pruning the consensus mammalian tree in [24]. The MTE hypothesis assumes that mass-specific BMR scales with body mass with an exponent of −0.25 and this was the case in our sample (95% CI of the slope: −0.26 < b < −0.19; n = 149).

Having established that the sample was suitable for testing the predictions of the MTE, we carried out three more regression analyses in the same R-packages. In all the analyses, the independent variable was log(10)(adult body mass). The dependent variables were log(10)(gestation duration), log(10)(lactation duration) and log(10)(total development time), respectively. We used PGLS regression to test the relationship between body mass and life history durations for each individual order as well as the entire dataset. For all three durations we examined whether the 95% confidence intervals of our allometries encompassed the predicted value of 0.25.

Recently Borries et al. [25] have questioned the accuracy of the primate life history data included in PanTHERIA. It is undoubtedly the case that PanTHERIA could be better. For example, it is not clear that species’ values are fully comparable across all the variables. We therefore repeated our gestation duration analyses using the three datasets that Borries et al. [25] suggest are more accurate than PanTHERIA, and the results we obtained are indistinguishable from those reported below. We also repeated the primate lactation duration analysis using a carefully screened dataset [17], and the results of that analysis were not different from the results reported below either. Details of these extra analyses are provided in Additional file 1.

## Results

**Figure 1:**
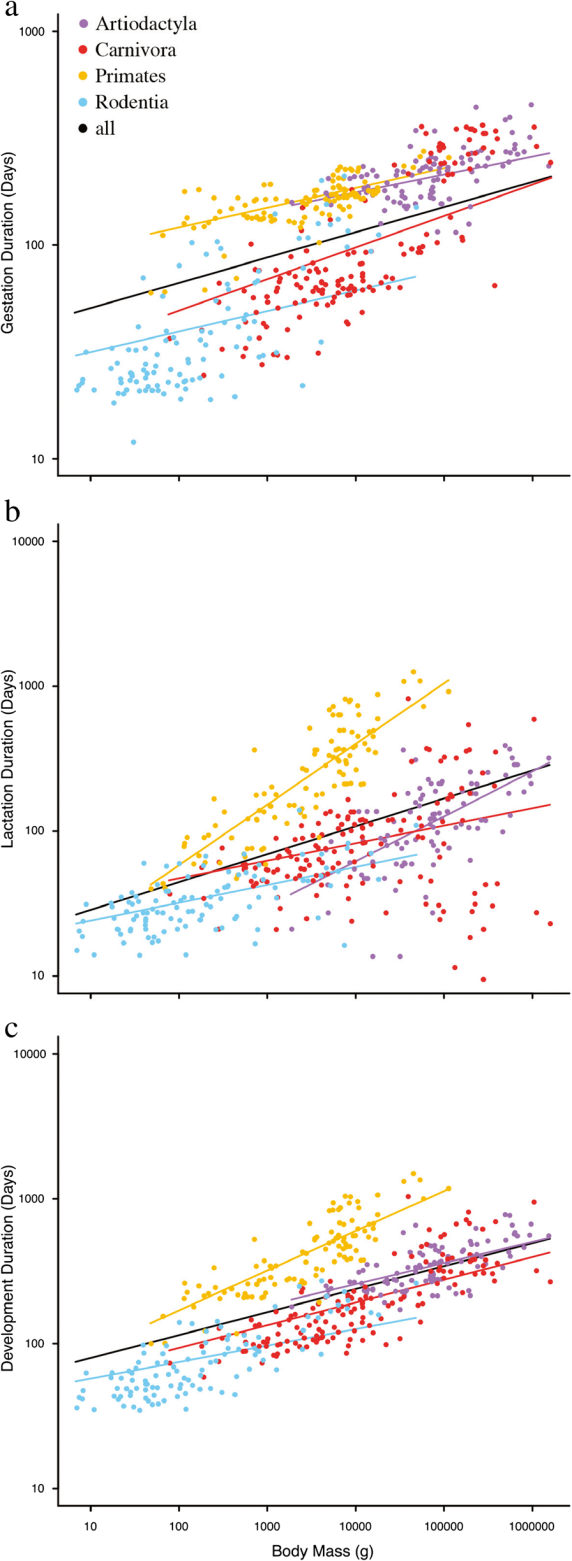
**Plots of (A) gestation duration, (B) lactation duration, and (C) total development time** ***vs*** **adult body mass for 457 mammalian species.** Durations in days. Body masses in grams. Each point represents a species mean. Note log-transformed axes. Regression lines are the phylogenetically-controlled best fit lines calculated using PGLS.

The results of the PGLS analyses are summarized in Table 1 and Figures 1a-c. Estimated phylogenetic signal for the three traits was high. Measured using Pagel’s lambda, signal ranged from 0.95 < λ < 0.99 for gestation duration, 0.57 < λ < 0.88 for lactation duration and 0.87 < λ < 0.95 for development duration. All orders have allometric slopes for gestation duration that fall below the 0.25 slope predicted by the MTE when analysed separately (Figure 1a). Carnivores have a steeper gestation slope (0.10 < b < 0.20) than the other three orders, which have overlapping slopes (Artiodactyla: 0.05 < b < 0.11; Primates and Rodentia: 0.06 < b < 0.13). Primates and artiodactyls have near identical and relatively high y-intercepts [(58 < a < 106) and (54 < a < 122) respectively], while rodents (18 < a < 35) and carnivores (14 < a < 46) have overlapping, lower y-intercepts; these intercepts are consistent with the longstanding observation that primates and artiodactyls are mostly precocial while rodents and carnivores are mainly altricial. The allometric slope for gestation also falls below the 0.25 slope predicted by the MTE when the orders are analysed together (0.10 < b < 0.14).

**Table 1:** **95% confidence intervals of phylogenetically-controlled allometric slopes and de-logged intercepts (Y = aM** ^**b**^ **) of gestation, lactation, and total development time in number of days**

		**Gestation**		**Lactation**		**Development**	
**Order**	**N**	***b***	***a***	***b***	***a***	***b***	***a***
Artiodactyla	100	0.05 - 0.11	54 - 122	0.23 - 0.4	1 - 10	0.11 - 0.19	41 - 110
Carnivora	146	0.10 - 0.20	14 - 46	0.04 - 0.2	10 - 72	0.11 - 0.20	27 - 75
Primates	106	0.06 - 0.13	58 - 106	0.33 - 0.51	4 - 18	0.21 - 0.34	29 - 80
Rodentia	105	0.06 - 0.13	18 - 35	0.08 - 0.17	14 - 24	0.08 - 0.15	34 - 57
All	457	0.10 - 0.14	28 - 53	0.15 - 0.23	11 - 31	0.13 - 0.18	41- 75

The allometric slopes for lactation duration are more variable than those for gestation duration (Figure 1b). The slopes for carnivores (0.04 < b < 0.20) and rodents (0.08 < b < 0.17) fall below the value predicted by the MTE; the artiodactyl slope is consistent with the MTE (0.23 < b < 0.4); and the primate slope is steeper than the MTE-predicted value (0.33 < b < 0.51). Artiodactyla has the lowest y-intercept (1 < a < 10) and overlaps with Primates (4 < a < 18) and Carnivora (10 < a < 73). Carnivora has the highest y-intercept, overlapping with both Primates and Rodentia (14 < a < 24). When all orders are analysed together, the allometric slope for lactation duration is close to, but still shallower, than the slope predicted by the MTE (0.15 < b < 0.23), and the intercept is intermediate in value (11 < a < 31).

Primates alone show a total development time allometry that is consistent with the predictions of the MTE (0.21 < b < 0.34). The total development time allometries for Artiodactyla (0.11 < b < 0.19), Carnivora (0.11 < b < 0.20) and Rodentia (0.08 < b < 0.15) overlap closely, but all fall below the MTE predicted value. The y-intercept confidence intervals for total development time for the four orders also overlap (Table 1). The scaling exponent obtained for total development time when all species are analysed together falls below the value predicted by the MTE (0.13 < b < 0.18).

## Discussion

The results of our analyses do not support the MTE. Only primates have a total development time scaling exponent that is consistent with the predictions of the MTE. The other three orders all have total development times that scale with body mass with a substantially shallower exponent of 0.15. The durations of the individual components of total development time—gestation and lactation—also do not scale with body mass in the manner predicted by the MTE. None of the orders has a gestation duration allometry that conforms to the predictions of the hypothesis; they all have gestation duration allometries that are shallower than predicted by the MTE. Only one order—Artiodactyla—has a lactation duration allometry that conforms to the predictions of the MTE. The lactation duration allometries for two of the other orders—Rodentia and Carnivora—are shallower than predicted by the MTE, while the lactation duration allometry for the fourth order—Primates—is steeper than predicted by the MTE.

The results of our analyses are consistent with those obtained by Dubman et al. [17] and Clauss et al. [18] but inconsistent with those obtained by Hamilton et al. [11]. There appears to be a simple explanation for this discrepancy: Clauss et al. [18] found that gestation duration scales with an allometric slope of 0.25 when OLS regression was used but was much shallower when they corrected for phylogenetic autocorrelation. This suggests that the cause of the conflict between our results and those of Hamilton et al. [11] is likely their decision not to correct for the effects of phylogeny.

To evaluate this potential explanation, we followed Hamilton et al. [11] and subjected our combined dataset to mixed-effects linear modeling with Order treated as a random taxonomic effect using the lme4 package [26]. The results were more consistent with the predictions of the MTE, especially in the analyses that focused on gestation duration and total development time (gestation duration: 0.20 < b < 0.24; lactation duration: 0.17 < b < 0.24; total development time: 0.24 < b < 0.25). That our dataset yielded results that are more consistent with the predictions of the MTE when analyzed with the method employed by Hamilton et al. [11] supports the idea that the discrepancy between our main results and Hamilton et al.’s results [11] is primarily due to the fact that we used full phylogenetic correction and they did not.

There are two reasons for thinking our slopes are more accurate than those reported by Hamilton et al. [11]. One is that, while there has been some debate concerning the relative merits of model I *vs* model II regression approaches in allometric analyses [27], it is now generally accepted that phylogenetic correction produces more accurate estimates of allometric slopes [28,29]. As illustrated by Clauss et al. [18], OLS models may miss underlying patterns, including grade effects where different taxonomic groups have the same slope but different intercepts.

The other reason for thinking our slopes are more accurate than those reported by Hamilton et al. [11] is an empirical one. We compared the log-likelihoods for the OLS model (λ = 0) and PLGS model (λ = ML), and the log-likelihoods for the former were consistently lower than the log-likelihoods for the latter, and the chi-square comparisons were all significant (p < 0.05). This indicates that the PGLS estimates provide a significantly better data fit than the OLS estimates for our sample, and therefore support the contention that the slopes we obtained are more likely to represent the true allometric slopes for the three maternal energetic investment durations than the slopes reported by Hamilton et al. [11].

The departure of allometries for total development time and its individual components from the predictions of the MTE has serious implications for at least one of two key claims of the MTE—that energy transfer is constrained by the structure of the internal resource distribution networks, and that natural selection has maximized the rate of resource transfer within the body. The 0.15 exponents for total development time in Artiodactyla, Carnivora, and Rodentia indicate that total development times are more alike in small and large species of these orders than is predicted by the MTE. Assuming that infants are the same relative size at weaning, this means either that large species develop for a shorter time, or that small species develop for a longer time, than the MTE predicts (Figure 1c). If durations are shorter at large body sizes, the implication is that large mammalian mothers transfer energy faster than they should be able to do so according to the MTE. This in turn implies that the structure of the internal resource distribution networks does not constrain energy transfer in the manner averred by the MTE. Conversely, if durations are longer than expected at small body sizes, this implies that, contrary to what the MTE contends, natural selection has not maximized energy transfer rates at small body sizes. Thus, the total development time allometries indicate that one or other of the claims is incorrect.

Using the same logic, the departure of allometries for gestation duration from the predictions of the MTE also indicates that at least one of the core claims of the MTE is incorrect. The fact that the gestation duration allometries for all four orders are shallower than predicted by the MTE could mean that large species develop *in utero* for less time than the MTE predicts, and therefore transfer energy via the placenta faster than they should be able to do so according to the MTE. That is, large mammals may have a higher growth rate compared to small mammals [18]. Alternatively, it could mean that small species develop longer *in utero* than the MTE predicts, and therefore transfer energy via the placenta slower than expected based on BMR, which in turn implies that natural selection has not maximized the rate of within-body energy transfer in smaller species resulting in relatively slower growth rates.

The implications of the lactation duration allometries for the MTE seem to be even more serious. The shallower-than-predicted allometries for Rodentia and Carnivora can be explained using the same logic as for gestation—either large-bodied mammals are transferring energy faster than predicted by the MTE, or small-bodied mammals are not transferring energy at the maximum possible rate, contrary to the MTE. In contrast, the steeper-than-predicted allometry for primate lactation indicates that either large species develop *ex utero* for longer than the MTE predicts, and therefore transfer energy via the mammary glands slower than they should according to the MTE, or small species develop for less time *ex utero* than the MTE predicts, and therefore transfer energy via the mammary glands faster than the MTE suggests they should be able to do. Viewed together, the lactation duration allometries raises the possibility that *both* the claim that energy transfer is constrained by the structure of the internal resource distribution networks *and* the claim that natural selection has maximized the rate of resource transfer may be wrong.

Dubman et al. [17] proposed a revised version of the MTE hypothesis in light of the allometries they obtained for primates. Because they found that total development time scales as predicted by the MTE, but gestation duration and lactation duration do not, Dubman et al. [17] suggested that gestation duration and lactation duration in primates are coupled traits evolving under the constraint of BMR such that species can trade-off the lengths of gestation and lactation but have to do so within a total development time dictated by BMR. This “coupled-traits hypothesis” is also not supported by our data. While primate total development time scales with the expected 0.25 exponent, the other three orders all have total development times that scale with body mass with an exponent of 0.15. So, the proposed BMR constraint on total development time does not hold for Artiodactyla, Carnivora, and Rodentia. More problematically still, our data also do not support the trade-off part of the coupled-traits hypothesis. The allometries of gestation duration and lactation duration are more variable across the four orders than the allometries for total development time, as expected under a trade-off scenario. However, the coupled-traits hypothesis predicts a negative relationship between the residuals of the allometry of lactation duration and the residuals of the allometry of gestation duration, and this prediction is also not supported by our data. The relationships between the two sets of residuals are generally positive rather than negative, but highly variable across the orders, with the only strong relationship being in the Primates (0.12 < b < 1.24; n = 106; Artiodactyla: −0.01 < b < 0.98; n = 100; Carnivora: −0.24 < b < 0.3; n = 146; Rodentia: −0.02 < b < 0.42; n = 105). Thus, it appears that the coupled-traits hypothesis is also not a good explanation for the variation in the duration of the key components of the mammalian maternal energetic investment cycle.

The failure of our analyses to support the predictions of both the original MTE hypothesis and Dubman et al.’s [17] modified version has some important implications for future research on the factors governing energetic investments in offspring by mammalian mothers. One is that it suggests the influence of BMR on gestation duration and lactation duration is more complicated than suggested by the MTE and coupled-traits hypotheses. Metabolic rate must constrain gestation duration and lactation duration in some way, because gestation and lactation involve the transfer of energy and therefore can only be afforded once demands of the mother’s basal metabolism have been satisfied. But our results indicate that the constraint must be indirect rather than direct. Another important implication for future research on the factors governing energetic investments in offspring by mammalian mothers stems from our finding that gestation duration and lactation duration allometries are so different one from the other within and across orders. This suggests not only that we need to allow for the possibility that different factors drive gestation duration and lactation duration, but also that we must take into account the possibility that the drivers of variation in the two durations differ among clades.

With regard to future work, total development time and its components are not the only biological times whose scaling exponents have been found to be inconsistent with the predictions of the MTE. Several others can be identified in the recent literature. For example, Duncan et al. [30] investigated the scaling of age at first reproduction in 1197 species of mammal, and obtained an exponent that is significantly lower than the 0.25 predicted by the MTE. Similarly, Müller and colleagues [31] examined food-in-gut retention time in 77 herbivorous mammals and found that it does not scale with an exponent of 0.25. Lemaître et al. [19] provide a third example. These authors examined the scaling of longevity in a sample of 1213 mammalian species, and also obtained a scaling exponent that was significantly different from 0.25. The fact that several, diverse biological times do not scale in the manner predicted by the MTE clearly raises questions about the MTE’s general applicability. It would seem, then, that an important task is to determine whether the MTE’s failure to predict scaling exponents for biological times is matched by a failure to predict scaling exponents for other types of trait. Another worthwhile undertaking would be to investigate the statistically indistinguishable slopes and intercepts for total development time in Artiodactyla, Carnivora, and Rodentia. That the slopes and intercepts for total development time are the same for three such diverse orders is intriguing. Could it be that their development is in fact constrained by a common factor, just not by basal metabolic rate?

## Conclusions

The present study demonstrates that the MTE’s prediction that mammalian maternal investment durations should scale with body mass with an exponent of 0.25 is not supported by the four best-studied mammalian orders—primates, carnivores, rodents, and artiodactyls. Thus, the influence of basal metabolic rate on mammalian maternal investment durations must be more complicated than the MTE envisages. Other factors must play an important role. Future research needs to allow for the possibility that different factors drive gestation duration and lactation duration, and that the drivers of the two durations may differ among orders.

## Endnote

^a^We recognize that “Artiodactyla” is no longer universally accepted as the name for the even-toed ungulate order. We use it here in order to be consistent with the source of the data we employed, the PanTHERIA database [20].

## Additional file

Additional file 1:
**Additional file for Jackson et al.’s ‘Basal metabolic rate and maternal energetic investment durations in mammals’.**
