# Supplementary material for: Basal metabolic rate and maternal energetic investment durations in mammals
Source: BMC Evol Biol. 2014 Sep 14;14:194. doi: 10.1186/s12862-014-0194-z (PMC4177257; doi:10.1186/s12862-014-0194-z)
Supplement: Additional file 1: — Additional file for Jackson et al.’s ‘Basal metabolic rate and maternal energetic investment durations in mammals’. [file 12862_2014_194_MOESM1_ESM.pdf]

Additional file for Jackson et al.'s 'Basal metabolic rate and maternal energetic investment durations in mammals'

Recently Borries et al. (2013) have questioned the accuracy of the primate life history data included in PanTHERIA. To address this concern, we repeated our gestation duration analyses using the three datasets that Borries et al. (2013) suggest are more accurate than PanTHERIA: Ernest (2003), Kappeler and Pereira (2003) and Rowe and Myers (2014). We also repeated the primate lactation duration analysis using a carefully screened dataset (Dubman et al., 2012).

Results of the PGLS regressions are summarized in Table S1, and relationships between datasets are shown in Figure S1. Though there are individual discrepancies (as pointed out by Borries et al., using a much smaller range of body sizes), the 95% confidence intervals for the phylogenetically-corrected primate gestation slopes were nearly identical across datasets (all between $0.05 > m > 0.14$). Primate y-intercepts also fell within a narrow range, $4.04 > b > 4.7$. Our dataset and Ernest's dataset showed carnivore slopes and intercepts as nearly identical ($2.6 > m > 3.8$ and $2.7 > m > 3.8$; $0.10 > b > 0.20$ and $0.10 > b > 0.19$). There also were no differences in the allometries of primate lactation duration for our dataset and Dubman et al.'s dataset. Thus, none of the alternative datasets returned a slope or intercept significantly different from our dataset. In other words, the datasets preferred by Borries et al. (2013) also do not support the MTE hypothesis.

References cited

Borries, C., Gordon, A.D., & Koenig, A. 2013 Beware of primate life history data: a plea for data standards and a repository. *PLoS ONE* **8**, e67200. (DOI 10.1371/journal.pone.0067200)

Dubman, E., Collard, M. & Mooers, A.Ø. 2012 Evidence that gestation duration and lactation duration are coupled traits in primates. *Biol. Lett.* **8**, 998-1001. (DOI 10.1098/rsbl.2012.0642)

Ernest, S.K.M. 2003 Life history characteristics of placental nonvolant mammals. *Ecology* 84, 3402.

Kappeler, P.M. and Pereira, M.E. 2003 *Primate life histories and sociology*. University of Chicago Press.

Rowe, N., and Myers, M. (eds.) (2014) www.alltheworldsprimates.org. Primate Conservation Inc. Accessed February 2014.

Table S1. 95% confidence intervals of allometric slopes and intercepts for gestation duration obtained from four different life history data sets with PGLS.

Order	This study	Ernest	Kappeler & Pereira	Rowe & Myers	Dubman et al.
Carnivora	n=147<br>*b*=0.10-0.20<br>*a*=2.6-3.8	n=135<br>*b*=0.10-0.19<br>*a*=2.7-3.8	-	-	-
Primates	n=106<br>*b*=0.06-0.13<br>*a*=4.1-4.7	n=93<br>*b*=0.06-0.13<br>*a*=4.1-4.7	n=88<br>*b*=0.06-0.11<br>*a*=4.2-4.7	n=84<br>*b*=0.05-0.14<br>*a*=4.04-4.6	n=66<br>*b*=0.05-0.12<br>*a*=4.1-4.7

Figure S1. Plots showing relationships between this study's dataset and alternative datasets. A) This study's primate gestation dataset vs Dubman et al.'s primate gestation dataset. B) This study's primate gestation dataset vs Ernest's primate gestation dataset. C) This study's primate gestation dataset vs Kappeler and Pereira's primate gestation dataset. D) This study's primate gestation dataset vs Rowe and Myers' primate gestation dataset. E) This study's carnivore gestation dataset vs Ernest's carnivore gestation dataset. F) This study's primate lactation dataset vs Dubman et al.'s primate lactation dataset.

A)

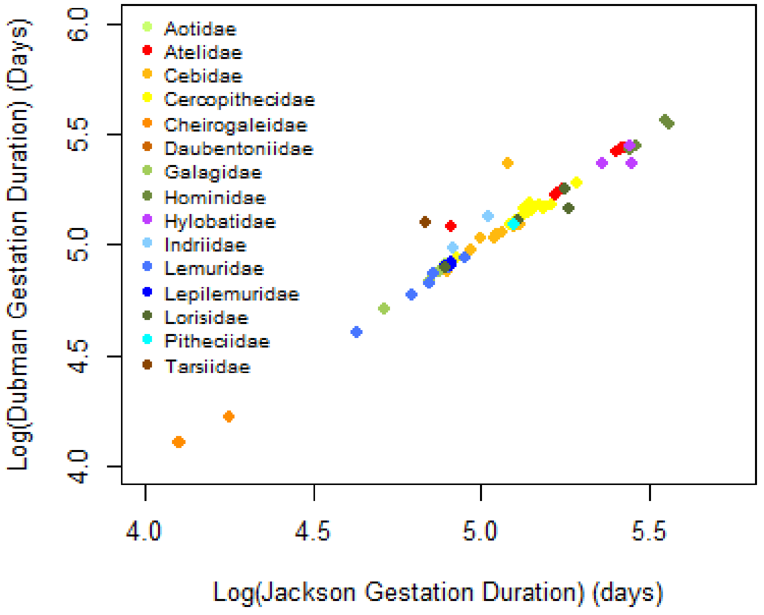

B)

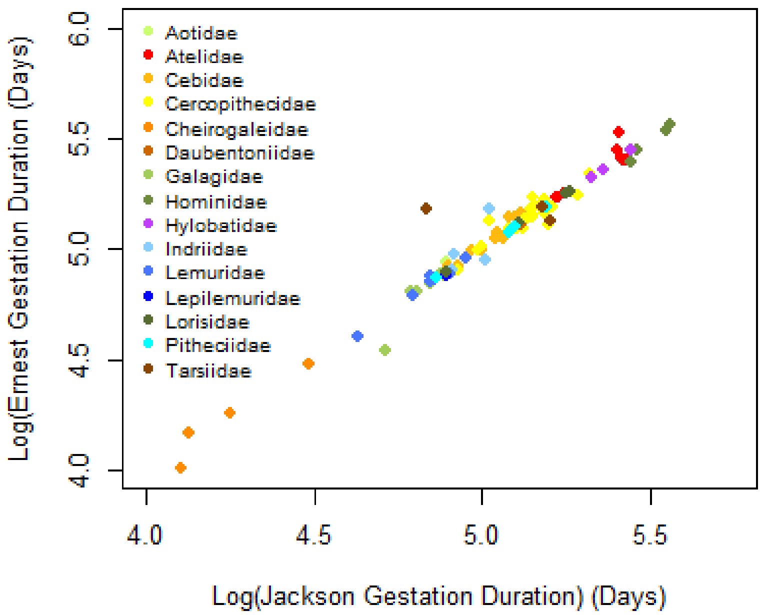

Aotidae
Atelidae
Cebidae
Cercopithecidae
Cheirogaleidae
Daubentoniidae
Galagidae
Hominidae
Hylobatidae
Indriidae
Lemuridae
Lepilemuridae
Lorisidae
Pitheciidae
Tarsiidae
Log(Ernest Gestation Duration (Days)
Log(Jackson Gestation Duration) (Days)
4.0
4.5
5.0
5.5
6.0

C)

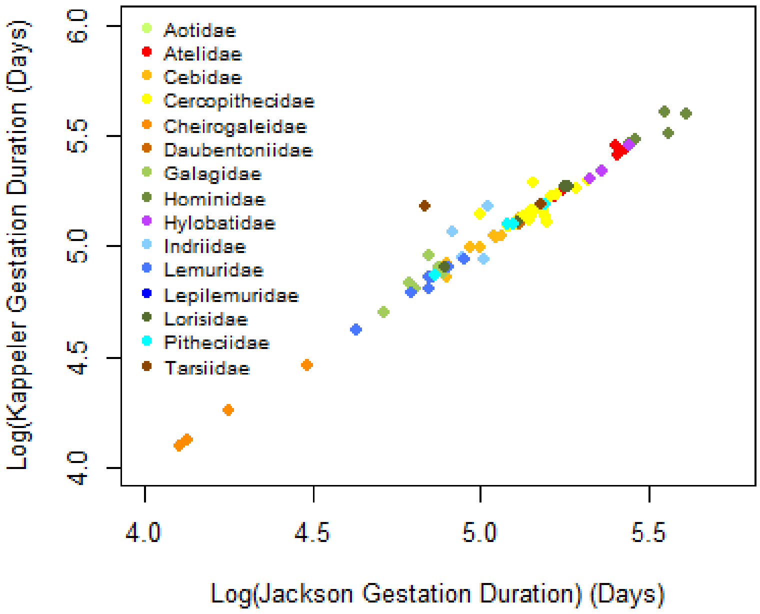
Aotidae
Atelidae
Cebidae
Cercopithecidae
Cheirogaleidae
Daubentoniidae
Galagidae
Hominidae
Hylobatidae
Indriidae
Lemuridae
Lepilemuridae
Lorisidae
Pitheciidae
Tarsiidae
Log(Kappeler Gestation Duration (Days))
4.0
4.5
5.0
5.5
6.0
4.0
4.5
5.0
5.5
Log(Jackson Gestation Duration) (Days)

D)

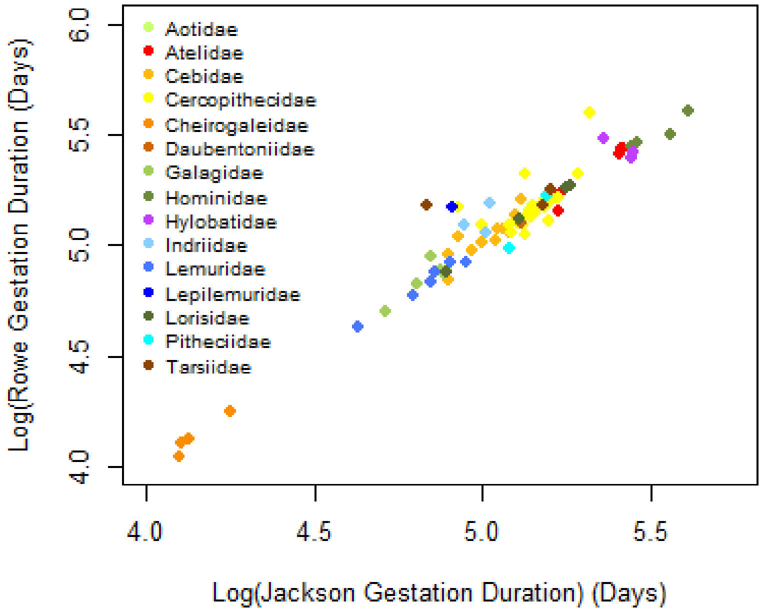
Aotidae
Atelidae
Cebidae
Cercopithecidae
Cheirogaleidae
Daubentoniidae
Galagidae
Hominidae
Hylobatidae
Indriidae
Lemuridae
Lepilemuridae
Lorisidae
Pitheciidae
Tarsiidae
Log(Rowe Gestation Duration (Days))
4.0
4.5
5.0
5.5
6.0
Log(Jackson Gestation Duration) (Days)

E)

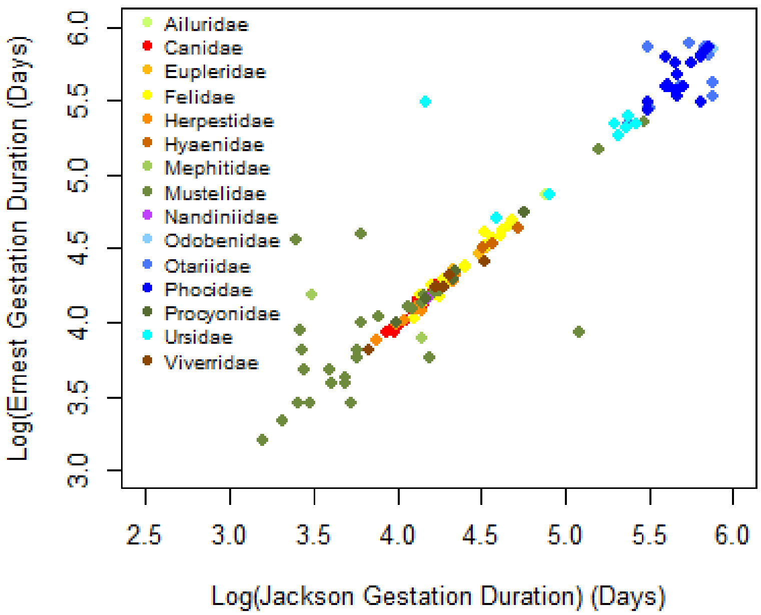
Ailuridae
Canidae
Eupleridae
Felidae
Herpestidae
Hyaenidae
Mephitidae
Mustelidae
Nandiniidae
Odobenidae
Otariidae
Phocidae
Procyonidae
Ursidae
Viverridae
Log(Ernest Gestation Duration (Days)
3.0
3.5
4.0
4.5
5.0
5.5
6.0
2.5
3.0
3.5
4.0
4.5
5.0
5.5
6.0
Log(Jackson Gestation Duration) (Days)

F)

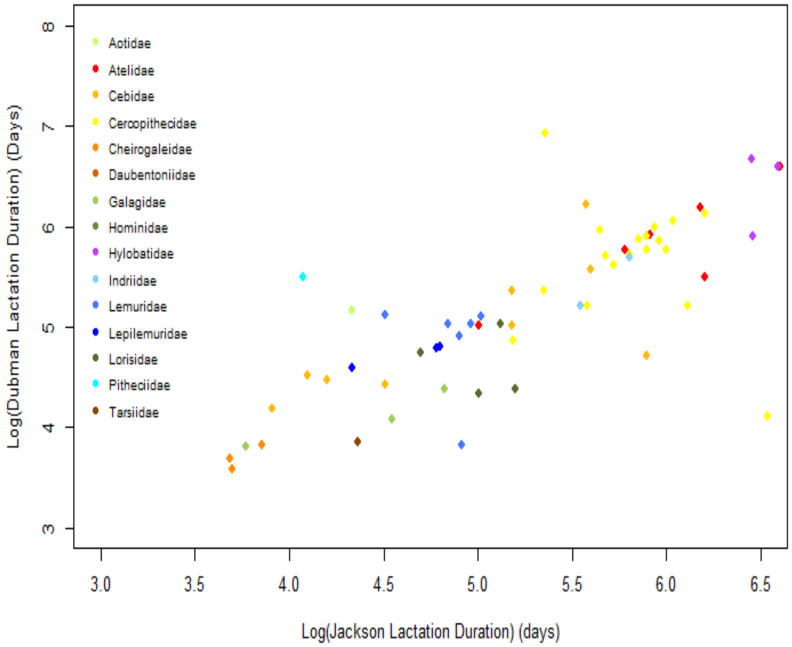

Aotidae
Atelidae
Cebidae
Cercopithecidae
Cheirogaleidae
Daubentoniidae
Galagidae
Hominidae
Hylobatidae
Indriidae
Lemuridae
Lepilemuridae
Lorisidae
Pitheciidae
Tarsiidae
Log(Dubman Lactation Duration) (Days)
Log(Jackson Lactation Duration) (days)
3.0
3.5
4.0
4.5
5.0
5.5
6.0
6.5
3
4
5
6
7
8
